# Terlipressin in the treatment of hepatorenal syndrome: A systematic review and meta-analysis

**DOI:** 10.1097/MD.0000000000010431

**Published:** 2018-04-20

**Authors:** Haiqing Wang, Aixiang Liu, Wentao Bo, Xielin Feng, Yong Hu

**Affiliations:** Department of Hepato-Biliary-Pancreatic Surgery, Sichuan Cancer Hospital & Institute, Sichuan Cancer Center, School of Medicine, University of Electronic Science and Technology of China, Chengdu, China.

**Keywords:** adverse event, hepatorenal syndrome, hepatorenal syndrome reverse, terlipressin

## Abstract

**Background:**

Hepatorenal syndrome is a fatal complication of advanced cirrhosis. Terlipressin is the most widely used treatment method, however, the therapy effects remain inconsonant. We aim to systematically assess the safety and efficacy of terlipressin for hepatorenal syndrome.

**Methods:**

We conducted a systematic review and meta-analysis. Randomized controlled trials involving terlipressin for hepatorenal syndrome were included in a systematic literature search. Two authors independently assessed the studies for inclusion and extracted the data. A meta-analysis was conducted to estimate the safety and efficacy of terlipressin for hepatorenal syndrome.

**Results:**

A total of 18 randomized controlled trials including 1011 patients were included. Hepatorenal syndrome reverse rate was 42.0% in the terlipressin group and 26.2% in the non-terlipressin group. Terlipressin had greater hepatorenal syndrome reverse rate and renal function improvement rate than placebo and octreotide in the management of HRS. Comparing to norepinephrine, terlipressin had similar efficacy, but with more adverse events. No significant difference of the efficacy was found between terlipressin and dopamine treatment. The subgroup analysis for type 1 HRS had the above same results, except that the adverse events were not significant different between norepinephrine group and terlipressin group.

**Conclusions:**

Terlipressin was superior to placebo and octreotide for reversal of hepatorenal syndrome and improving renal function, but it had no superiority comparing to norepinephrine.

## 1 Introduction

Hepatorenal syndrome (HRS) is a fatal complication of advanced cirrhosis with ascites and liver failure, with nearly 50% of patients dying within 2 weeks after the onset.^[1]^ HRS is now recognized as a form of renal failure that occurs as the consequence of the interplay between various hemodynamic changes in patients with advanced cirrhosis.^[2]^ The peripheral arterial vasodilatation hypothesis holds that splanchnic and systemic arterial vasodilatation in end-stage liver disease results in reduction in the effective circulating volume. In response to the reduction in circulating volume, the systemic endogenous vasoconstrictor systems are activated and effective arterial underfilling happened. These processes culminate in renal vasoconstriction and hepatorenal syndrome happened.^[3,4]^ Studies have suggested that HRS is functional abnormality in the kidneys and is a potentially reversible syndrome.^[2]^ Therapy with systemic vasoconstrictors and albumin is an effective option to ameliorate renal dysfunction and to improve survival.^[5]^ Terlipressin is the most widely used vasoconstrictor in the world. Several randomized controlled trials (RCTs)^[6–13]^ have evaluated the therapeutic effect of terlipressin and found it is more effective in improving renal function in patients with hepatorenal syndrome, comparing to other drugs or placebo. However, other RCTs^[14–19]^ found that terlipressin had no advantage on improving renal function than other drugs or placebo. So, the therapy effects of terlipreesin for HRS are inconsonant and we conducted this systematic review and meta-analysis to evaluate the safety and efficacy of terlipressin for patients with HRS.

## 2 Materials and methods

### 2.1 Systematic literature search

Two authors independently conducted a systematic literature search of electronic databases including the Cochrane Central Register of Controlled Trials, Embase, Science Citation Index (Web of Knowledge), and PubMed up to June 11, 2017. The search strategies were as follows: (“terlipressin” OR “ glypressin” OR “vasoconstrictor agents”) AND “hepatorenal syndrome.” The literature search was performed with restriction in language to English and types of studies to RCTs. The completed search results were merged by using Endnote X4 (reference management software) and duplicate records were removed. Titles and abstracts of the references identified were scanned by 2 independent authors. If compliance with inclusion criteria was not clear from the abstract, we retrieved the full text for further assessment. The study protocol was approved by the Clinical Research Ethics Committee of Sichuan Cancer Hospital & Institute, Sichuan Cancer Center, School of Medicine, the University of Electronic Science and Technology of China conformed to the ethical guidelines of the 1975 Declaration of Helsinki. No written informed consent was obtained from all patients because this study was a meta-analysis.

### 2.2 Inclusion and exclusion criteria

*Types of studies.* Only RCTs were considered for inclusion in this review. Other types of studies such as nonrandomized controlled trials, historical controlled trials, cohort studies, and case–control studies were excluded.

*Types of participants.* Patients who were diagnosed as HRS according to the criteria of International Ascites Club^[20]^ and the updated criteria in 2007^[21]^ were included in our study, irrespective of the types of HRS.

*Types of interventions*. Our meta-analysis included comparisons of terlipressin alone or with albumin versus placebo, albumin, or other vasoconstrictors. These trials comparing terlipressin with transjugular intrahepatic portosystemic shunts, dialysis, and liver transplantation were excluded.

*Types of outcome measures*. The primary outcomes of our study were HRS reverse, renal function change and mortality. The secondary outcomes were HRS recurrence and adverse events. HRS reverse was defined as a decrease in serum creatinine to 133 μmol/L (1.5 mg/dL).^[7,8,14]^ Renal function change was defined as a 50% serum creatinine decreasing from baseline but with a final value >133 μmol/L (>1.5 mg/dL).^[7,8]^ Recurrence of HRS defined as increase in serum creatinine >1.5 mg/dL in patients with HRS reverse.^[16]^

### 2.3 Data collection and analysis

Any disagreement during study selection and data extraction was resolved by discussion and referral to a third author for adjudication. Two authors extracted data on a standard form that included population characteristics, terlipressin dosage, and the outcome measures in each trial. In the case of missing data, we contacted the original investigators to request further information.

### 2.4 Assessment of methodology quality

Two authors assessed the methodological quality of the trials independently and the Jadad score^[22]^ was used to assess the quality of RCTs, with a cumulative score of >4 indicating high quality.

### 2.5 Statistical analysis

We pooled the synchronized extraction results as estimates of overall treatment effects in the meta-analysis using Review Manager for Windows version 5.3 (The Cochrane Collaboration, Oxford, England). The estimated effect measures were risk ratio (RR) for dichotomous data and weighted mean difference (WMD) for continuous data; both reported with 95% confidence interval (CI). We checked all results for clinical and statistical heterogeneity. Clinical heterogeneity was evaluated by assessing study populations and interventions, definition of outcome measures, concomitant treatment, and perioperative management. Heterogeneity was determined using the χ^2^ test with significance set at *P *= .05 and *I*^2^ statistics were used for the evaluation of statistical heterogeneity (*I*^*2*^ ≥ 50% indicating presence of heterogeneity). We used a fixed-effects model to synthesize the data when heterogeneity was absent; otherwise a random-effects model was used for synthesizing the data. Data were presented as forest plots and the funnel plot was used to assess publication bias.^[22]^ Sensitivity analyses were carried out by including RCTs only with high quality.

## 3 Results

### 3.1 Description of included trials

**Figure 1:**
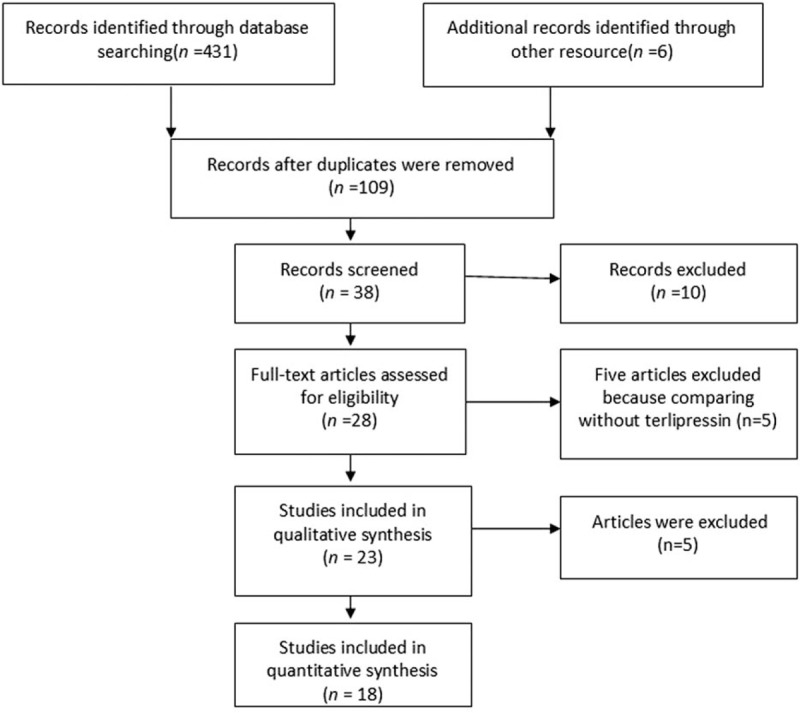
Flow diagram of the study selection process.

A total of 431 articles were initially yielded by our literature search and 6 additional records identified through other resource. After excluding the other articles, 23 RCTs were further identified. Four repeated articles and one single-arm article were excluded, and at last 18 RCTs^[6–19]^ including 1011 patients (509 patients in the terlipressin group and 502 patients in control group) met the criteria for inclusion in the meta-analysis (Fig. 1). These articles published between 2001 and 2016, with the sample size from 15 to 196 patients. The characteristics of all the RCTs and included patients information were summarized in Tables 1 and 2. All the patients were diagnosed as hepatorenal syndrome based on the International Ascites Club criterion.^[20,21]^ Ten trials^[8–10,13–15,19,23–25]^ only included type 1 HRS patients and three RCTs^[12,16,17]^ only included type 2 HRS, the other 5 studies^[6–7,11,18,26]^ had both types. The dosages of terlipressin were different among the RCTs, ranging from 1.5 to 12 mg/day depending on the patients’ condition. Twelve of the trails maintained a maximum terlipressin treatment duration of 2 weeks, but 2 studies^[11,18]^ only had 5 days’ treatment and 4 RCTs^[12,19,25,26]^ did not describe the duration. Seven RCTs^[7–12,27]^ compared the efficacy and safety between terlipressin and placebo, 8 RCTs^[14–17,19,23,24,26]^ compared between terlipressin and norepinephrine, 1 RCT^[25]^ compared terlipressin with octreotide, 1 RCT^[12]^ compared terlipressin with dopamine and the last RCT^[6]^ compared terlipressin with octreotide and midodrine. The methodological quality of all the included trials was displayed in Table 3. According to the Jadad score, 11 of the 18 RCTs were considered as high quality with the score >4.

**Table 1:**
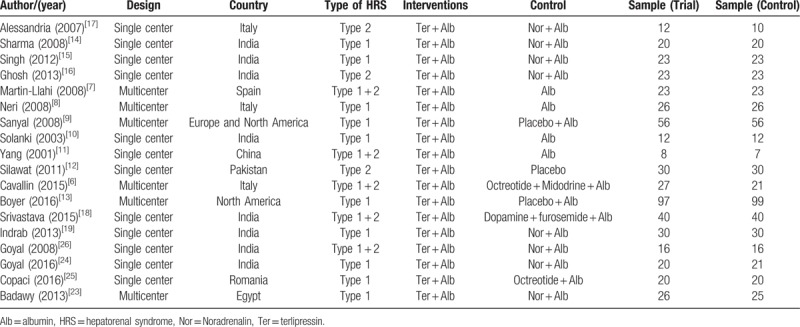
Characteristics of the included randomized controlled trials.

**Table 2:**
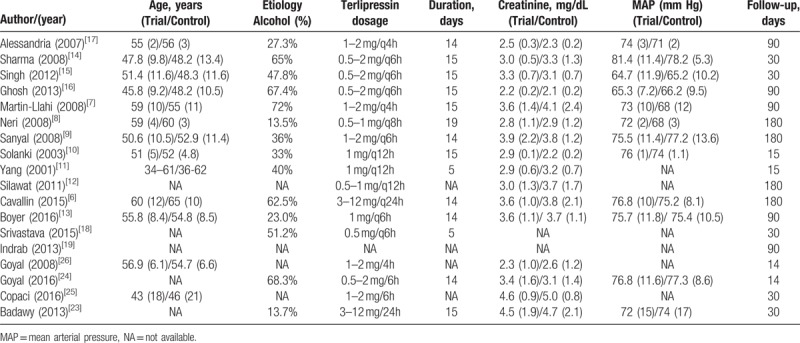
Characteristics of the patients included in these randomized controlled trials.

**Table 3:**
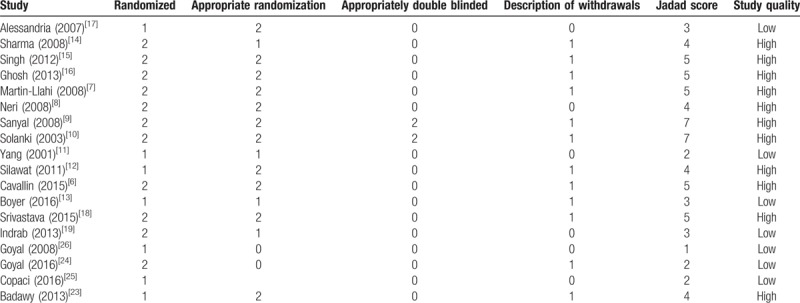
Quality assessment of the included randomized controlled trials based on the Jadad scoring system.

### 3.2 Comparisons the efficacy and safety between terlipressin and placebo

#### 3.2.1 Hepatorenal syndrome reverse

**Figure 2:**
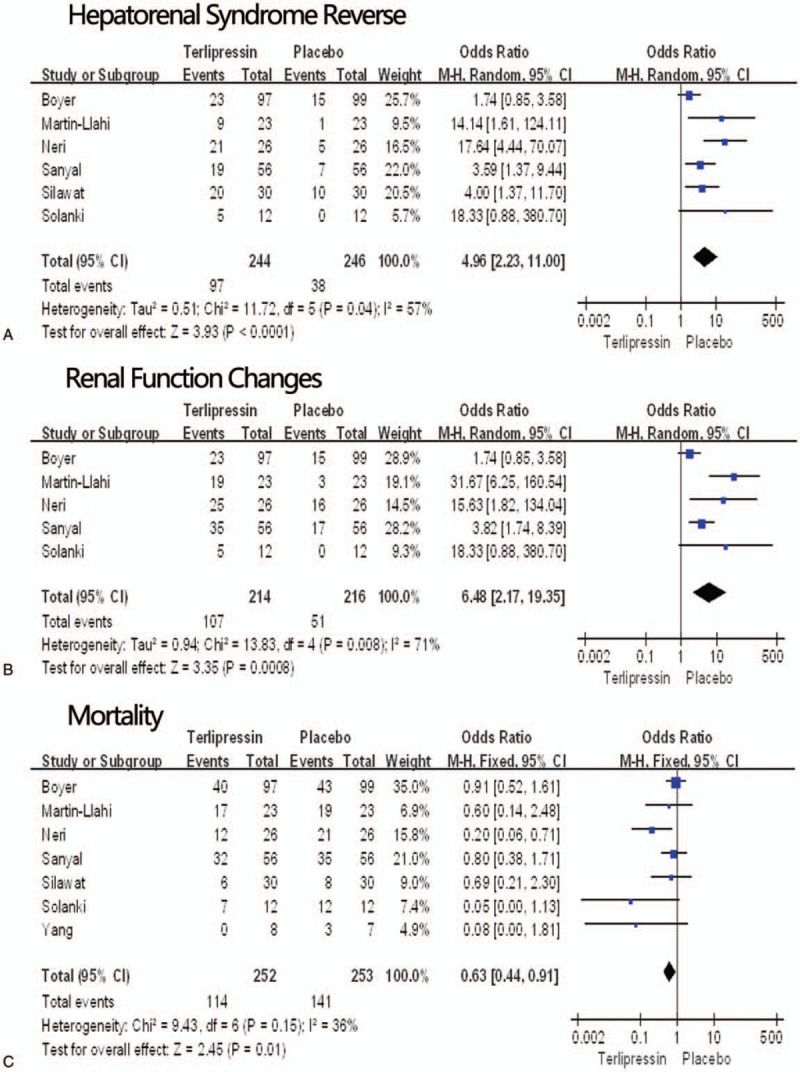
Hepatorenal syndrome reverse. (A) Renal function changes (B), mortality (C) in the terlipressin group versus the placebo group. CI = confidence interval, M-H = Mantel–Haenszel, P = probability.

Six of the seven RCTs^[7–10,12,27]^ reported the event of hepatorenal syndrome reverse. In the included studies, the rates of hepatorenal syndrome reverse were 39.8% in the terlipressin group and 15.4% in the placebo group. The meta-analysis showed that terlipressin group had greater hepatorenal syndrome reverse rate than placebo group (4.96, 95%CI: 2.23−11.0, *P *= .001, *I*^2^ = 57%) (Fig. 2A).

#### 3.2.2 Renal function change

Renal function change was observed in 5 studies^[7–10,27]^ and the total rate was 44.4%. 50.0% of the patients in terlipressin group and 23.6% in the placebo group achieved renal function change. Our meta-analysis indicated that the risk ratio for renal function change with terlipressin therapy was 6.48 (95% CI 2.17−19.35; *P* = .0008) times than the placebo group (Fig. 2B).

#### 3.2.3 Mortality

Seven RCTs^[7–12,27]^ reported the mortality, with the follow-up duration from 15 days to six months. The mortality ranged from 0% to 79.1%, with the overall rate of 45.2% in the terlipressin group and 55.7% in the placebo group. Meta-analysis found that terlipressin group had less deaths than the placebo group (RR = 0.63, 95%CI: 0.44–0.91, *P* = .01, I2 = 36%) (Fig. 2C).

#### 3.2.4 Hepatorenal syndrome recurrence and adverse events

Only 1 RCT^[9]^ involved the recurrence of HRS and the meta-analysis found that there was no significant difference between terlipressin group and placebo group. In these included RCTs, the adverse events were with no uniform definition and were reported based on different standards, such as severe adverse events, drug-related adverse events, total complications and so on. The most common reported adverse events for terlipressin group were abdominal cramps, arrhythmia, and cyanosis of the toe.^[2,4]^ Three RCTs^[9,11,27]^ reported adverse events and the overall rate of 55.9% in the terlipressin group and 41.4% in the placebo group. Our meta-analysis showed no significant difference between the 2 groups (RR = 1.57, 95%CI: 0.63–3.93, *P* = .33, *I*^2^ = 60%).

### 3.3 Comparisons the efficacy and safety between terlipressin and norepinephrine

#### 3.3.1 Hepatorenal syndrome reverse

**Figure 3:**
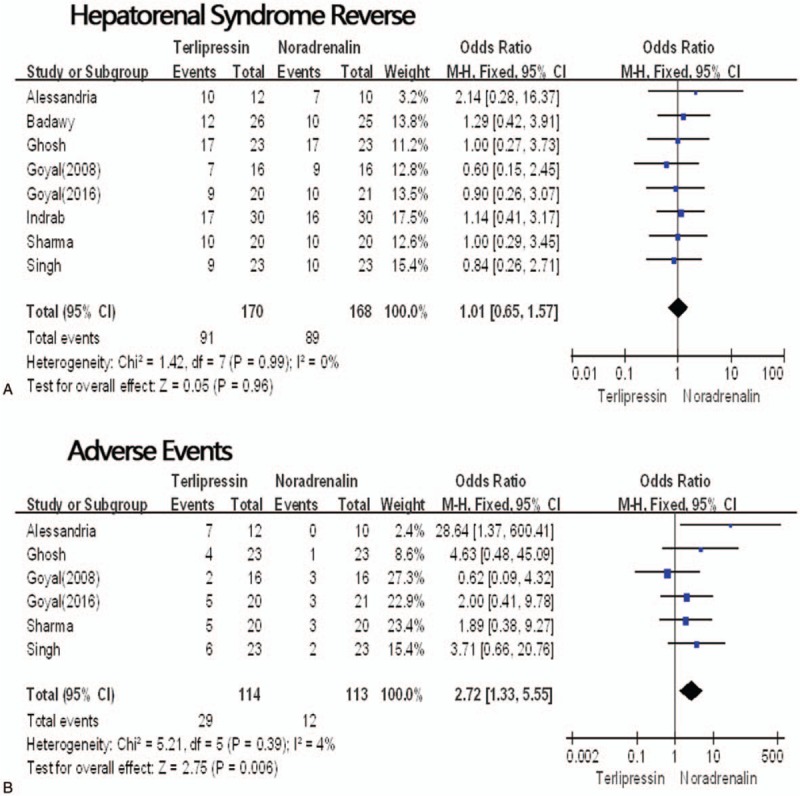
Hepatorenal syndrome reverse. (A) Adverse events (B) in the terlipressin group versus the norepinephrine group. CI = confidence interval, M-H = Mantel–H aenszel, P = probability.

All the 8 RCTs^[8,14,16,17,19,23,24,26]^ reported the hepatorenal syndrome reverse, the hepatorenal syndrome reverse rates ranged from 41.3% to 77.3%, with the overall rate of 53.5% in the terlipressin group and 52.9% in the norepinephrine group. The meta-analysis showed no significant difference between the 2 groups (RR = 1.01, 95%CI: 0.65–1.57, *P* = .96, *I*^2^ = 0%) (Fig. 3A).

#### 3.3.2 Renal function change

Renal function change was reported in 4 studies.^[14–17]^ Around 60.3% of the patients in terlipressin group and 61.8% in the norepinephrine group achieved renal function improvement. However, our meta-analysis indicated that no significant difference was found between the 2 groups (RR = 0.91, 95% CI: 0.46–1.79, *P* = .79, *I*^2^ = 0%).

#### 3.3.3 Mortality

Only 1 RCT^[26]^ did not report the mortality. The mortality ranged from 31.8% to 95%, with the overall rate of 61.7% in the terlipressin group and 62.0% in the norepinephrine group. Meta-analysis found that no significant difference was found between the 2 groups (RR = 1.05, 95%CI: 0.63–1.74, *P* = .86, *I*^2^ = 0%).

#### 3.3.4 Hepatorenal syndrome recurrence and adverse events

Three RCTs^[16–17,19]^ showed the hepatorenal syndrome recurrence and six RCTs^[14–17,24,26]^ reported adverse events. But hepatorenal syndrome recurrence rates were not different by our meta-analysis (RR = 0.51, 95%CI: 0.19–1.33, *P* = .17, *I*^2^ = 19%). The total adverse events were 25.4% in the terlipressin group and 10.6% in the norepinephrine group, thus terlipressin had more adverse events (RR = 2.72, 95%CI: 1.33–5.55, *P* = .006, *I*^2^ = 4%) (Fig. 3B).

### 3.4 Comparisons the efficacy and safety between terlipressin and other vasoactive drugs

One RCT^[25]^ investigated the difference between terlipressin and octreotide, and found that renal function improved was observed in both groups. However, terlipressin group had more hepatorenal syndrome reverse rate than the octreotide group (55% vs 20%, *P* = .01), the hepatorenal syndrome recurrence and adverse events were not reported. Terlipressin comparing with dopamine was studied by another RCT.^[18]^ Twenty-four-hour urine output and plasma renin activity were improved in both terlipressin and dopamine group, and the 2 group had similar one-month mortality considering HRS-1 and HRS-2. Only one RCT^[6]^ compared terlipressin with the combination of octreotide and midodrine. The study found that terlipressin plus albumin is significantly more effective than midodrine and octreotide plus albumin in reversal of renal failure (55.5% vs 4.8%, *P* < .001) and improving renal function (70.4% vs 28.6%, *P* = .01) in patients with HRS.^[6]^

### 3.5 Subgroup analysis for type 1

HRS with Type 1 had worse prognosis than type 2, so we carried out subgroup analysis for the types of HRS-1 including 10 RCTs.

#### 3.5.1 Comparisons between terlipressin and placebo for HRS-1

Four RCTs^[8–10,27]^ comparing terlipressin with placebo with 384 patients only included type 1 HRS. Our meta-analysis found that terlipressin group had higher hepatorenal syndrome reverse rate (RR = 4.92, 95%CI: 1.60–15.09, *P* = .005, *I*^2^ = 70%), higher renal function change rate (RR = 3.77, 95%CI: 1.54–9.27, *P* = .004, *I*^2^ = 52%) than placebo group, but the hepatorenal syndrome recurrence, mortality and adverse events were similar between the 2 groups.

#### 3.5.2 Comparisons between terlipressin and norepinephrine for HRS-1

For type 1 HRS subgroup, terlipressin and norepinephrine had similar hepatorenal syndrome reverse rate (RR =0.93, 95%CI: 0.54–1.60, *P* = .78, *I*^2^ = 0%), renal function change rate (RR = 0.75, 95%CI: 0.32–1.77, *P* = .51, *I*^2^ = 0%), mortality and adverse events.

### 3.6 Publication bias

The funnel plot was based on mortality, renal function change rate, and hepatorenal syndrome reverse. As no study was outside the limits of the 95% CI, there was no evidence of publication bias.

## 4 Discussion

Although HRS is a functional syndrome, it is still associated with a rapid deterioration of multiple organ function and a poor prognosis, especially for type 1 HRS.^[21]^ Liver transplantation is the best treatment of choice for HRS, but both the short life expectancy of HRS and the worldwide organ shortage limits this therapy method.^[28]^ Many treatment methods can be used for hepatorenal syndrome, such as vasoconstrictors and albumin, transjugular intrahepatic portosystemic stent-shunt, and extracorporeal albumin dialysis, but vasoconstrictors is the most widely used therapy method because of its therapeutic effect and convenience.^[4]^ Simultaneously, vasoconstrictors also serve as a bridge wait for liver transplantation. Terlipressin is the most effective and widely used vasoconstrictor. It can not only reduce portal inflow and thereby decrease portal pressure, but also reduce the extent of the systemic vasodilatation, leading to a rise in the systemic arterial blood pressure, which in turn will improve the renal perfusion pressure^[2]^ and renal function.

The present review suggested that terlipressin had superiority in improving both hepatorenal syndrome reverse rate and renal function comparing to placebo and octreotide in the management of HRS. The efficacy of management of HRS between terlipressin and norepinephrine was similar, but terlipressin had more adverse events. Terlipressin also had similar survival for hepatorenal syndrome comparing with dopamine, but the effect of hepatorenal syndrome reverse and renal function improvement was not compared. In summary, terlipressin was superior to placebo and octreotide for reversal of hepatorenal syndrome and improving renal function, but it had no superiority comparing with norepinephrine.

The total hepatorenal syndrome reverse was 42.0% in the terlipressin group in our meta-analysis, which was higher than the control group with the rate of 26.2%. The result was similar with other studies^[5,28,29]^ with reported rates from 46% to 58.9%. However, the hepatorenal syndrome reverse in the included RCTs of the meta-analysis ranged from 23.7% to 80.7%. Many studies have proved that HRS type 1, nonalcoholic liver disease as etiology, high model for end-stage liver disease score and high baseline serum creatinine influenced hepatorenal syndrome reverse.^[30,31]^ In our meta-analysis, we also found HRS types and serum creatinine affected the therapeutic effect of terlipressin. HRS 1 patients only achieved 32.1% of HRS reverse, but HRS 2 got as high as 72.3% of HRS reverse. Meanwhile, the RCTs^[8,16–17]^ with the first three high HRS reverse rates >70% had lower serum creatinine level < 2.8 mg/dL, and the remaining RCTs with HRS reverse rates <70% all had pretreatment serum creatinine >2.9 mg/dL. In our subgroup analysis for type 1 HRS, we found that terlipressin benefited of HRS reverse and renal function improvement compared with placebo, but it had similar efficacy and safety with norepinephrine. This suggested that terlipressin had stronger ability to improve renal function than the placebo and some vasoconstrictors, but it was not superior to norepinephrine.

Another result with significant difference between terlipressin (25.4%) and norepinephrine group (10.6%) was adverse events. But the subgroup analysis with HRS-1 found that terlipressin and norepinephrine had similar adverse events. Three reasons could explain this result. Firstly, the terlipressin dosage was large in the studies with HRS-2 patients. One^[23]^ of the 5 studies only with HRS-1 had a maximal 24 terlipressin dosage of 12 mg, but 2^[17,26]^ of the remaining 3 studies (with HRS-2) had a maximal 24 terlipressin dosage of 12 mg. The large terlipressin dosage maybe increase adverse events. Secondly, advanced analysis found that there were few available data to analysis on adverse events in these RCTs. Thirdly, in the RCTs with reported adverse events, most studies only reported terlipressin specificity complications, such as abdominal cramps and arrhythmia, which necessarily added the adverse events of terlipressin because the placebo and norepinephrine barely had the specificity complications. For example, Alessandria et al^[17]^ reported 7 adverse events in the terlipressin group and zero in the norepinephrine group. Ghosh et al^[16]^ reported 4 adverse events (2 abdominal cramps, 1 arrhythmia and 1 cyanosis of the toe) in the terlipressin group and one even in the norepinephrine group. On the other hand, the most reported complications were usually self-limiting and nonfatal. Even so, caution should be taken before the management of terlipressin and monitoring of adverse events is still essential.

Before our review, several meta-analysis and system reviews had been carried out. Several studies^[28,29,32,33]^ only compared terlipressin with albumin and Nassar et al^[5]^ only compared terlipressin with norepinephrine in his meta-analysis. Zhang et al^[34]^ and Gluud et al^[35]^ included both terlipressin and other vasoconstrictor drugs for HRS, but the included RCTs were published before 2010. A latest meta-analysis^[36]^ compared terlipressin with placebo and other vasoconstrictor drugs was published on 2017, but it did not compared terlipressin with placebo and did not include the all RCTs.^[24,26]^ Four new meta-analysises^[37–40]^ compared the influence of different vasoactive drugs for the treatment of hepatorenal syndrome, but not just terlipressin. There was no updated meta-analysis to evaluate the effect of terlipressin for HRS. Several new RCTs^[23–26,41,42]^ on terlipressin have been search, so we conducted this meta-analysis. But a one-armed RCT^[42]^ and one duplication RCT^[41]^ (part of Neri's RCT^[8]^) were exclude in our meta-analysis. Different from other meta-analyses, our study had the advantage of including all the related and new RCTs and comparing terpipressin with all the other drugs. Similarly, our study had the same conclusion with the others. All the previous meta-analysis^[5,28,29,31–34]^ involving HRS reverse found that terlipressin increased the number of patients with reversal of HRS as well as adverse events.

The quality of the evidence in our study was relative high because we only included RCTs. Although some methodological weakness existed in the RCTs, such as allocation concealment, lack of sample size calculations, and lack of blinding, Eleven of the 18 RCTs were of high quality with a Jadad score >4. In order to reduce bias, we conducted a subgroup analysis by including RCTs with HRS-1, the results still suggested that terlipressin could increase the number of patients with reversal of HRS and could improve the renal function.

Some limitations still existed in our review. Firstly, the sample sizes were small in the RCTs and most of them had no sample size calculations. Two studies^[10,17]^ had patients <24 and only 2 RCTs^[9,13]^ had sample size >100. The limited sample volume may weaken the strength of the evidence. Secondly, less data was available for adverse events, which did not benefit of safety evaluation for terlipressin. Meanwhile, only 3 RCTs focused on patients with type 2 HRS and the conclusion on this type HRS was not analyzed. Thirdly, the heterogeneity of the patients in the included trials may have influenced the conclusions because some trials diagnosed HRS using the previous criterion^[20]^ and some using the updated criterion.^[21]^ So, more RCTs with strict methodology and large sample size should be conducted to further evaluate the terlipressin.

In conclusion, this review provided the best available evidence for the safety and efficacy evaluation of the terlipressin for treating HRS. On the basis of this evidence, terlipressin could improve both hepatorenal syndrome reverse rate and renal function comparing to placebo and other vasoconstrictor drugs except norepinephrine in the management of HRS, with acceptable more drug-related adverse events. Terlipressin and norepinephrine had similar effect for HRS.

## Author contributions

**Conceptualization:** Haiqing Wang, Aixiang Liu, Yong Hu.

**Data curation:** Aixiang Liu, Wentao Bo, Xielin Feng.

**Investigation:** Haiqing Wang, Aixiang Liu, Yong Hu.

**Methodology:** Aixiang Liu, Wentao Bo, Xielin Feng, Yong Hu.

**Software:** Xielin Feng.

**Writing – original draft:** Haiqing Wang.

**Writing – review & editing:** Xielin Feng, Yong Hu.
